# Medical gazes and meteorological metaphors: representations of dementia in contemporary motion pictures

**DOI:** 10.1186/s13584-018-0283-3

**Published:** 2019-02-20

**Authors:** Mark Schweda

**Affiliations:** Division of Ethics in Medicine, Department of Health Services Research, University of Oldenburg, Ammerländer Heerstr. 114-118, 26129 Oldenburg, Germany

**Keywords:** Dementia, Film, Media representations, Ethics

## Abstract

**Background:**

For the last two decades, there has been a surge of major motion pictures dealing with the topic of dementia. This trend reflects and at the same time informs and shapes an increasing public awareness of dementia as an individual ethical and sociopolitical issue.

**Main body:**

This contribution examines from an ethical perspective how contemporary motion pictures deal with the topic of dementia and thus contribute to forming our moral awareness of the phenomenon as such and our ways of dealing with those affected. It focuses on an analysis of the conceptual premises and aesthetic imagery relevant in the cinematographic representation of dementia. As the analysis will show, viewing a film as a film about dementia may presume a medicalizing perspective. Furthermore, cinematographic images and metaphors are powerful devices for articulating thoughts and feelings about the elusive and ultimately ineffable experience of dementia. At the same time, however, they can also have problematic implications.

**Conclusions:**

Given the lack of knowledge and widespread fear, stigmatization and exclusion, health policy can and should use cinematographic approaches in order to enhance public understanding of dementia and empathy with those affected. At the same time, however, public health agencies and educational institutions making use of the persuasive power of film need to pay closer attention to the images and messages constructed, their aesthetic functioning and their ethical implications and social consequences.

## Background

For the last two decades, we have witnessed a surge of major motion pictures dealing in one way or the other with the topic of dementia: sophisticated biopics like *Iris* (Richard Eyre, UK/USA 2001), crime thrillers like *The Memory of a Killer* (Erik Van Looy, Belgium 2003), dramas like *Away from Her* (Sarah Polley, Canada 2006) or *The Savages* (Tamara Jenkins, USA 2007), but also science fiction blockbusters like *Rise of the Planet of the Apes* (Rupert Wyatt, USA 2011), light-hearted crime capers like *Robot and Frank* (Jake Schreier, USA 2012) or dark comedies like *Mita Tova* (Tal Granit, Israel 2014).

It seems plausible to assume that this is not just some historically accidental cumulation, but rather a symptomatic trend pertinent to the general “dementia boom” [1] in contemporary popular culture: a growing public awareness of dementia as an individual and sociopolitical issue, comprising the increasing epidemiological incidence, the growing number of people directly or indirectly affected, the desperate search for a cure, horror visions of a forgetful aging society. Sometimes “dementia” almost seems to become a metaphor for our late-modern times in general, the “signature disease” of the twenty-first century in which the aging societies of our day and age seem to recognize characteristic features of their own psychosocial and sociocultural state and development [2].

On the other hand, film as a mass medium not only reflects public awareness like a mirror. It also has the potential to shape this awareness: to draw our attention to new developments, to sensitize us for occurring moral or political problems, to frame issues in a certain way, even to shape our perception and our emotional attitudes towards them, thus influencing our way of dealing with things. Just speaking from the perspective of bioethics, it is well known how Miloš Forman’s *One Flew over the Cuckoo’s Nest* (USA 1975) drew public attention to problems of traditional authoritarian psychiatry, thus playing a pivotal role in popularizing the concerns of the anti-psychiatry-movement [3]. We also know how Michael Crichton’s *Coma* (USA 1978) focused and formulated a widespread uneasiness with organ donation, creating iconic images for common fears surrounding the developing organ transplantation system [4]. Or how Jonathan Demme’s mainstream movie *Philadelphia* (USA 1993) helped to promote sympathy for and acceptance of people with HIV/AIDS among the general public [5].

Of course, this “transformational” potential of film to provide information, influence views and attitudes, and raise medicine and healthcare issues to the public agenda, has not gone unnoticed in the academic and political sphere. In fact, it is located at the core of approaches of "entertainment education" in health communication that have evolved since the 1950s [6]. The essential idea is to design communication strategies involving mass media messages that both entertain and educate, thus using entertainment to raise awareness, improve knowledge, and induce behavioral and social change [7]. Entertainment education has been implemented and developed all around the world, giving rise to a variety of theoretical and methodological approaches. Examples comprise the employment of radio programs, soap operas, and telenovelas as well as feature movies to address issues of domestic violence and safer sex in India or Latin America, HIV/AIDS prevention in Africa, or breast and prostate cancer in the US (for an overview, see [8]). In recent years, one notable area of application has been the fight against stereotypes and stigmatization in the context of mental illness [9].

Against this backdrop, the contribution at hand explores from an ethical point of view how recent motion pictures deal with moral issues in the context of dementia (some of the following considerations were initially developed in [10]). The leading question is in which way these pictures might actually contribute to forming our moral awareness of the phenomenon. The main line of argument focuses on recent Anglo-American and continental movies dealing with dementia and only refers to a handful of selected exemplary scenes. But when is a film actually a film about dementia? The first section considers this seemingly obvious question before the second goes on to examine the cinematographic language that is employed to visualize issues of dementia, taking a closer look at one of the most dominant kinds of dementia images which could be termed “meteorological imagery”. Finally, this example of “meteorological imagery” will be used to discuss ethical implications of the cinematographic staging of dementia for public perceptions of the phenomenon as such and the ways of treating affected persons, also drawing conclusions for the potentials and problems of using popular movies in public health communication in the context of dementia.

### Medical gazes – When is a film actually a film about dementia?

In the biopic *The Iron Lady* (UK/France 2011), starring Meryl Streep as former British prime minister Margaret Thatcher, the frame narrative shows Thatcher as an old woman looking back on significant stages of her life and political career. Even before the movie came out, it had already sparked intense public and political controversy: Fellow members from Thatcher’s conservative party found it distasteful and degrading that their great icon seemed to be depicted as a senile and disturbed old woman [11]. And critics from the political left objected – conversely – that Thatcher was shown in a softening, humanizing light, promoting sympathy and blinding out the more disagreeable aspects of her political persona [12]. Either way, the consensual underlying conviction in both camps was that the film shows Thatcher as a person with dementia, that it is actually a film dealing with dementia.^1^

Against this backdrop, it appears at least remarkable that the movie itself actually never even makes any explicit mention of dementia. No one ever uses the word or similar expressions. The protagonist is not clinically diagnosed or openly addressed as having dementia. Even in a scene that plays in a doctor’s office after a medical examination, no clear statements about Thatcher's state of health are made. In fact, this very scene eventually culminates in a rhetorically elaborate monologue of the former prime minister on the superior power of thought over human existence. As we will see, this already points to the central problem: We ultimately cannot even understand the films basic dramaturgic premise and “message” properly as long as we interpret it in the medical terms of dementia.

There are at least three scenes commentators refer to in claiming the film shows a Margaret Thatcher suffering from dementia. The first one is the opening scene, introducing the old Margaret of the frame narrative. It shows an elderly woman shopping for milk in a grocery store. She appears a bit insecure and out of touch with present day business and hustle: She hesitates regarding the wide range of dairy products in the refrigerated shelves. She is obviously offended by the discourteous behavior of a young customer. When she sees the newspaper headline on Islamist terror attacks, she seems to have difficulties to classify the events. And upon learning at the counter what a pint of milk costs, she reacts with disbelief.^2^

Another scene from the movie's first minutes shows Thatcher at the breakfast table vis-a-vis her husband Denis. The two of them have what seems like a longtime couple’s casual dialogue over breakfast, discussing the rising prices of dairy products and quipping about possibly having to economize and sublet a room. However, when the household help enters the kitchen, we suddenly see the scenery from her perspective and realize that Margaret is actually sitting at the table alone, the implication being that her dialogue partner was nothing more than a figment of her own imagination. This scene provides the second and apparently most important evidence named for Thatcher’s dementia: She frequently talks to her recently deceased husband Denis. In fact, this turns out to be one of the central dramaturgic devices of *The Iron Lady*: Throughout the whole movie, we see the old Margaret Thatcher reviewing and commenting significant points in her former life and political career as well as her present situation in long conversations with her deceased spouse.

The third scene shows the former prime minister in a dialogue with her daughter Carol: She is sitting at the dressing table in her bedroom while Carol takes her to task about leaving the house alone. Thatcher reacts irritated and surly to these concerns and reproaches her daughter about having nothing better to do with her life than making a fuss about her old mother. In the background, we see Denis sitting on the bed with a towel turban around his head, completing a crossword puzzle and asking for a word with nine letters that describes the unwillingness to change course. In this scene, we see the old Margaret Thatcher displaying – and actually spelling out – the primary character trait repeatedly named as evidence for her dementia in the reviews: She is obstinate, stubborn and grumpy, and does not comply with her closer social surrounding’s expectations.

This sums up some of the evidence named for *The Iron Lady* being a film about dementia: Its protagonist is an old woman who is out of touch with present life, talks to her dead husband and behaves in an erratic and grumpy manner. Regardless of how these points would be evaluated in a clinical diagnosis, dementia is by far not the only possible explanation. Some of the behavior in question actually appears quite understandable and sensible. Being out of touch is what can happen when older people disengage from active participation in public life [13]. Continuing the dialogue with the great love of one’s life, longtime companion and confidant, even after their death, is something many widowed people are reported doing and probably a healthy coping mechanism in the process of bereavement and grieving [14]. And being obstinate is not really a completely unfamiliar trait in Thatcher’s public personality, but rather the one quality she was often praised for during her active political career, a trait that actually helped earn her the title “Iron Lady” in the first place [15].^3^

Against this backdrop, the persistent classification of the film as a film about dementia is in need of explanation. On closer inspection, it actually might be much more significant for the perspective of the audience, the recipients themselves, than for the film’s own intrinsic intention, structure, and message. Thus, the reviews illustrate that the word “dementia” is no longer just a technical scientific term reserved for the medical profession. It has found its way into public discourse and everyday life where it seems to be used in a rather broad and sweeping way, applying to any kind of behavior in older people that may be perceived as strange and erratic. It seems plausible to take this as yet another symptom for the medicalization of aging frequently noted in social gerontology: The expansion of the “medical gaze”, the medical perspective and jurisdiction, into the realm of old age, describing its manifestations in medical terms and turning its peculiarities into pathologies [16].

Of course, one objection comes to mind immediately: It is commonly known from media reports that the real Margaret Thatcher *was* actually dealing with cognitive impairment in her later years [17]. Maybe it is this knowledge that informs our perspective on the movie and justifies our assumption that the protagonist is suffering from dementia, as well. Surely, this objection has its point. However, not only does the film itself not provide sufficient grounds for the dementia interpretation. Indeed, its fundamental dramaturgic premise and message as a cinematic piece of art cannot be adequately understood, at all, as long as we regard it in the medicalizing perspective of dementia. *The Iron Lady* simply does not make sense when viewed as a “dementia movie”. The protagonist is not really cognitively impaired. After all, the whole plot is recapitulated from her point of view. And at its heart is not a story of cognitive decline, but a psychological conflict: The old Margaret Thatcher is struggling with loss, the loss of her political power, her public splendor, her lifelong companion. The central drama is not about her *forgetting the past*, but rather about her *trying to let go of the past* in order to be able to live on in the present.

This conflict culminates in Margaret’s relationship to her deceased husband: On one hand, she clings to Denis as her most important lifelong contact person, companion and confidant. On the other, she knows she has to let go of him in order to sustain her sense of reality and live in the here and now. A recurring plotline is that she is supposed to sort out Denis’ old clothes and give them away to a charity. She makes several attempts, but never quite succeeds, until the very end, the film’s final “showdown”. In this core scene, we see Margaret eventually packing Denis’ things in a frantic overnight cleanup and ultimately saying goodbye to her deceased husband who vanishes in a white flash. The very last scene of the film then shows her in a quiet and peaceful mood in the kitchen of her house, rinsing a tea cup over the sink, listening to the birds outside and looking out the window at children playing in the street. She has let go of the burdens of the past and lives in the present now. “I won’t go anywhere today”, she tells her assistant.

### Meteorological metaphors – How is dementia depicted in the movies?

Regardless of the different interpretations of *The Iron Lady*, the question is how movies that explicitly and decidedly deal with dementia treat their subject matter. Clearly, in an essentially visual art form like film, imagery plays a constitutive role. In general, images have a particular relevance and significance whenever we are dealing with phenomena or experiences for which there is no established conceptual scheme. Their specific significance in this context results from transference: They draw an analogy between a subject matter at hand and some other area of reality which does not necessarily have any obvious relations to it, thereby connecting the unknown to the already known and thus creating a metaphorical context of interpretation in which we are able to form an understanding of the matter at hand [18].

The inner world of a person with advanced dementia arguably constitutes such an inaccessible, elusive dimension of reality. Indeed, films dealing with dementia often come up with a great variety of images, a whole “pictorial language” trying to capture how it must be like to be affected by the syndrome (for the following overview, see [19]). For example, there are “structural metaphors” employing complex ideas such as the “journey” or the “path into forgetfulness”, the “loss of self” or the “return to childhood”. There are also “orientational metaphors” alluding to spatial coordinates and directions such as the “decline” or “slide into darkness”. Finally, there are “ontological metaphors” describing the effects of dementia by reference to concrete entities such as “empty shells”. One rather dominant kind of imagery that can be found throughout many cinematographic representations of dementia could be called “meteorological imagery” since it draws its images for dementia from the sphere of weather and atmospheric phenomena: fog like in *Iris* (2001), snow in all its variations like in *Away from Her* (2007), rain, like in *Small World* (2012), or twilight, like in *The Notebook* (2004).

On one hand, this “meteorological imagery” makes reference to a broad and longstanding tradition of philosophical and theological thought which employs optical metaphors for cognitive processes [20]. Thus, already in Plato‘s theory of knowledge, cognition is described in terms of visual perception, as *seeing* the ideas, that is, the very essence of things, with the mind’s eye. Neoplatonic philosophy develops a whole vocabulary describing degrees of being and nothingness, truth and falsehood, in terms of light and darkness. Christian thought keeps this optical imagery and merely shifts the light source. Thus, in Augustin’s *Confessions*, true knowledge comes from as a form of higher, divine illumination. And these are not just some old notions outdated by scientific progress. Also in modern times, the whole epistemological discourse is outright obsessed with optical metaphors, starting from Descartes’ idea of *evidence* as a basic feature of true knowledge, a “clear and distinct” mental representation of things. Indeed, it is the modern age that has often been defined in terms of a whole philosophical program, movement and epoch using one prominent optical metaphor as its signature emblem: Enlightenment, explicitly setting itself apart from an allegedly “dark” medieval age. The optical imagery also pervades our everyday language and popular discourse, for example when we say something is “clear”, “apparent” or “obvious”, when we have an “insight”, or “something dawns on us”.

The meteorological imagery of dementia builds on this long standing tradition of optical metaphors and develops it further. Where truth is light and cognition is visual perception, declining cognition can be symbolized as the impairment of this visual perception by meteorological phenomena: fog hampering our view and making the sight of things increasingly diffuse; rain running down a window pane, letting the outside world appear blurred and unrecognizable; falling snow that slowly covers a landscape, hiding everything under a layer of white and thus making all objects indiscernible; clouds obscuring the sun and keeping away its light, thus dimming down the whole scenery and casting large shadows; the twilight at dusk in which objects begin to lose their clearly defined familiar shape and slowly sink into darkness. This meaning and function of meteorological imagery can be illustrated exemplarily with a scene from *Small World*, a German-French co-production from 2010 directed by Bruno Chiche and starring Gerald Depardieu and Anna Maria Lara. In this film, Depardieu plays an older man who suffers from memory loss. He gets increasingly detached from present day life and returns to the places of his childhood world. In one scene, the snow has set in and draws him outside one night: We see snowflakes falling in front of a window pane, maybe symbolizing a last barrier of the inner sense of personality and self-awareness against the diffusion of the drifting snow. In a counter-shot, we now look – together with the protagonist – through the window and into the darkness outside. His reflection in the window pane gets lost as soon as he steps outside. He is fascinated by the flying snowflakes, but at the same time, they distract him from seeing the real world around him. He loses orientation, the camera moving around him in circles. In the next shot, the snow flurry gets more intense. The protagonist is already outside the city, walking through a snow covered landscape that no longer shows any houses or other familiar discernible objects. Darkness now impedes eyesight, also for the viewer. The falling snow has the effect of covering the protagonist’s tracks so that he will eventually not be able to trace his way back home. He gets lost in the snow covered woods. Disoriented, isolated and freezing, he sinks to the ground.

### “Into the sunset” – Ethical implications of cinematic framings of dementia

Metaphors are important. They draw an analogy between a subject matter and some otherwise unrelated sphere of reality, suggesting that both are similar in a particular respect. Thus, they connect the unknown to the already known, helping us form a basic understanding of the matter in question, especially where we still lack clear concepts. At the same time, however, such images can also be problematic. The figurative sphere has its own internal structure and logic beyond the point of comparison, and this “excess of metaphorical meaning” [21] can superimpose itself on the literal sphere and suggest inadequate descriptions and conceptions. This distortive effect of metaphorical excess meaning might also be at work in meteorological imagery and its implications for our perception of dementia.

To illustrate this point, it is worth returning to Margaret Thatcher and one of her old political friends, former US-president Ronald Reagan. Both are not only widely regarded as figureheads of the neoliberal movement that radically changed the postwar global social and economic order up to this day [22]. They actually also shared a similar personal fate. When Reagan was diagnosed with Alzheimer’s in 1991, he issued a now famous farewell letter, saying goodbye to the American people in the double sense of withdrawing from the public eye, and at the same time expecting to lose sight of the real world, himself. It is commonly known that before Reagan went into politics, he had been a famous Hollywood actor from the 1930s to 60s, starring in over 60 movies. And indeed, in the final lines of his farewell letter, he makes a reference to this past in the film industry, employing a classic movie image to describe what now lies ahead: the “journey into the sunset”. The lines read: “I now begin the journey that will lead me into the *sunset of my life*. I know that for America there will always be a *bright dawn* ahead.” [23]. Here, Reagan proves once more a master of political communication. He picks up a classic image from Western movie finales, the cowboy riding into the sunset, with its allusions to all-American frontier-mythology of heading west and into a new tomorrow. At the same time, he blends this image with the meteorological imagery of dementia, the fading light at dusk, impeding visual perception and making things lose their shape until they vanish into darkness.

When Reagan died in 2004, his old friend Margaret Thatcher gave one of the eulogies at his funeral service.^4^ The speech honors the former president’s character and political virtues and pays tribute to his role and achievements during the Cold War era. At the end, Thatcher actually takes up the sunset motif from Reagan’s letter and adds yet another layer of meteorological imagery:

“For the final years of his life, Ronnie’s mind was *clouded by illness*. That *cloud* has now lifted. He is himself again – more himself than at any time on this earth. For we may be sure that the Big Fella Upstairs never forgets those who remember Him. And as the last journey of this faithful pilgrim took him *beyond the sunset*, and as *heaven’s morning broke*, I like to think – in the words of Bunyan – that ‘all the trumpets sounded on the other side’. We here still *move in twilight*. But we have one *beacon to guide us* that Ronald Reagan never had. We have his example.” [24]

Apparently, this passage is replete with meteorological metaphors. The cinematographic scenery of the journey into the sunset evoked in Reagan’s letter is amended by clouds and twilight and thus tied back to the political topos of the Cold War as a battle between light and dark, good and evil. At the same time, the meteorological metaphors of light and dark are interwoven with Christian ideas of heaven and earth, this world and the afterworld, death, resurrection and eternal life. They give the whole image a stronger religious layer of meaning and Reagan himself the aura of a spiritual leader and almost messianic savior.

When we see how cinematic imagery can thus pervade into real life and public discourse, the seemingly academic issue of metaphorical excess meaning becomes eminently practical: It might actually have ethical implications and consequences for the way we perceive dementia and treat those personally affected. With reference to the example just described, I would like to point out – hypothetically – two possible kinds of consequences:

Within the framework of meteorological imagery, dementia is primarily framed in terms of cognition: Truth is light, seeing is cognition, and meteorologically impeded sight represents cognitive decline. “His mind is clouded by illness”, as Thatcher has it, with the imagery of “roaming in twilight” and “beacons of light to guide us” further emphasizing the suggestion that dementia is ultimately all about losing cognitive orientation in the world. This cognitivistic framing reflects the general negative image of dementia in a “hypercognitive society” [25]: First of all, while the meteorological imagery focuses on cognitive processes and their impairment, other aspects are likely to be “overlooked”. Thus, manifestations of dementia in the physiological, emotional or social domain are rather neglected. As a result, the focus rests on the one area where dementia can hardly be described in other terms than as failure, decline and degeneration. Of course, no one denies that dementia is an essentially negative experience. But in the cognitivistic perspective, even minor positive aspects are likely to be ignored or marginalized, e.g., increased receptiveness on the level of sensual experience and delight or the discovery of a new emotional intimacy of relationships often reported from caring family members. In consequence, the one-sided deficit oriented perspective suggested by meteorological imagery might reinforce and promote overly negative images of dementia as just one long “journey into darkness”.

Meteorological imagery also has a tendency to depict dementia as an external force, an alien power that befalls a person from the outside: Clouds, snow, fog or darkness crawling over the affected persons’ minds, surrounding them and at the same time isolating them from the rest of the world. This externalization actually has the implication that dementia is separated from the affected person, herself. As Thatcher’s eulogy for Reagan indicates, such a perspective can have odd consequences. The idea that someone’s “mind is clouded by illness” suggests that it still remains inherently intact behind the cover of clouds or fog and thus can be restored as soon as this veil is removed. “[H]e is himself, again”, as Thatcher puts it in view of Reagan, once the “clouds” of dementia have “lifted”. The experience that dementia actually constitutes an inherent and irreversible change of personality, some would even say a disintegration of personhood itself, at least as we know it, cannot be expressed within this metaphorical framework. In consequence, the meteorological perspective might not be able to do justice to the affected persons’ internal condition and constitution. It might actually lure us into seeing and addressing not so much the person with dementia, herself, but instead rather the alleged “inner” “actual”, “proper” person supposed to be locked somewhere behind or within the “shell” of the “demented self”. Dementia then appears as some sort of deceptive façade, a veil hiding the real individual within. Measuring someone’s actual present state against the ideal image of some long lost person under these metaphorical premises, it is hard to imagine how our view on the actual person with dementia could not be colored by sentiments of disappointment, impatience, and even reproach.

## Conclusions: Ethical considerations and implications for health policy

Film can be a valuable form of dealing with new developments and experiences in the field of medicine and healthcare. With its multidimensional combination of visual, auditory and verbal strategies and its manifold cognitive as well as affective impacts, it has an unparalleled potential for informing and shaping public awareness and changing personal attitudes or even behaviors [26]. In consequence, motion pictures have been increasingly discovered as a didactical tool in medicine and bioethics [27] and as a powerful instrument in public health information and policy campaigns [28].

Cinematographic images and metaphors can be especially important to grasp elusive phenomena and articulate complex thoughts and feelings. This becomes particularly relevant when there is no established conceptual scheme of things as is the case with the subjective experience of mental health conditions and cognitive impairments such as dementia. Here, film can actually help laypeople, relatives and healthcare professionals to form and express an idea of what it may be like to be affected. Thus, given the common lack of knowledge and widespread fear, stigmatization and exclusion, health policy can and should use cinematographic approaches to enhance public understanding of dementia and empathy with those affected [10].

By appealing to affective and emotional levels of experience, cinematographic accounts can especially highlight non-medical aspects of and approaches to dementia and dementia care. At the same time, however, it is necessary for filmmakers, knowledge multiplicators and audiences to reflect the role the recipient perspective plays in viewing a film as a film about dementia and a character as a person with dementia. In particular, this can raise awareness for the medicalizing tendencies at work in an all too broad and undiscerning application of the dementia terminology, as well as for its limitative effects on the perception of situations and events. As the case of *The Iron Lady* shows, the “medical gaze” can effectively prevent us from understanding what is really going on with older people – in film as well as in reality [10].

Furthermore, we must be careful not to surrender all too readily and uncritically to the suggestive appeal of the figurative sphere. While dementia and dementia care are highly ambivalent and complex experiences, we saw that prominent media representations have a tendency to promote misleading and negative public understandings and attitudes [29, 30]. Therefore, public health agencies and educational institutions capitalizing on the persuasive power of cinematographic approaches need to dedicate closer attention to the images and messages constructed in media discourses and especially in mass media such as film, their functioning and implications, and their practical consequences. In this sense, there are ethical limits to the strategic exploitation of “oblique persuasion” in this context [31]. Instead, health communication has to pursue a decidedly reflective and critical approach that is sensitive to the implications of cultural metaphors and supports the detection and analysis of inaccurate and stigmatizing views and images of dementia and those affected. Indeed, studies in the context of mental health indicate that the fight against stigma may be more effective when the persuasive power of motion pictures is systematically combined and counterbalanced with educational supplements [9].

Finally, going beyond these critical considerations, we also have to pave the way for positive, constructive approaches. This means that we need to develop innovative strategies and provide sufficient resources for encouraging and promoting a more adequate consideration of dementia in popular motion pictures as well as in the mass media in general. Of course, the crucial question here is who gets to decide what an adequate consideration of dementia looks like. In the field of entertainment-education, the general problem of achieving consensus over the definition of desirable, “prosocial” contents in modern pluralistic societies has been discussed at length [31]. Regardless of the concrete outcomes, one minimal procedural condition definitely should be that those directly affected get to have a say in the discussion. This means that we have to consider possibilities of including directly affected people themselves in the political debate and consultation as well as in the campaign design [32]. This participatory approach is not only a normative requirement of policy making in modern liberal democracies that call for the inclusion of those affected in public deliberation and decision making processes. It also appears to be an obvious and viable way to explore new, alternative representations and eventually draw a richer, more comprehensive and multifaceted picture of living with dementia in the public sphere [33, 34].

## Footnotes

1 The controversy also attested to the enduring impact of Margaret Thatcher on the collective memory of the British public. After all, her active political career had already been over for 20 years at that point. Interestingly, a medical study had already shown in the 1980s that British dementia patients’ memories of Thatcher tended to be more vivid and persistant than those of any other prime minister in recent history and even the Queen. The authors conclude: “We can only say that somehow Mrs. Thatcher has given an item of knowledge to demented patients that they would otherwise have lacked: she reaches those parts of the brain other prime ministers could not reach” [35].

2 This is in fact quite allusive. Abolishing free school milk in the 1970s earned the then-education secretary the nickname „Thatcher the milk snatcher“. And in her days as prime minister, she often stressed that as a grocer’s daughter, she knew the price of a pint of milk, implying that she was still in touch with everyday life and the concerns of ordinary people.

3 Her public reply to those demanding a „U-turn“of her fiscal policies on a Tory congress in 1980 has almost become proverbial: „You turn if you want to; the lady’s not for turning!“([15], p. 195).

4 It is an odd sideline of this story that at that time, Thatcher herself had suffered several minor strokes and had been advised not to talk live in public, anymore, so that the speech had been videotaped months before, when Reagan was still alive.
